# Mild hyperthermia by MR-guided focused ultrasound in an ex vivo model of osteolytic bone tumour: optimization of the spatio-temporal control of the delivered temperature

**DOI:** 10.1186/s12967-019-2094-x

**Published:** 2019-10-24

**Authors:** Pauline C. Guillemin, Laura Gui, Orane Lorton, Thomas Zilli, Lindsey A. Crowe, Stéphane Desgranges, Xavier Montet, Sylvain Terraz, Raymond Miralbell, Rares Salomir, Sana Boudabbous

**Affiliations:** 1 Image Guided Interventions Laboratory (GR-949), Faculty of Medicine, University of Geneva, Geneva, Switzerland; 2 Radiation Oncology Division, University Hospitals of Geneva, Geneva, Switzerland; 3 Radiology Division, University Hospitals of Geneva, Geneva, Switzerland; 4 Equipe Chimie Bioorganique et Systèmes Amphiphiles, Institut des Biomolécules Max Mousseron, UMR 5247, Avignon Université, 84911 Avignon, France

**Keywords:** Adjuvant hyperthermia, Osteolytic tumours, MR-guided focused ultrasound, Temperature control

## Abstract

**Background:**

Magnetic resonance guided focused ultrasound was suggested for the induction of deep localized hyperthermia adjuvant to radiation- or chemotherapy. In this study we are aiming to validate an experimental model for the induction of uniform temperature elevation in osteolytic bone tumours, using the natural acoustic window provided by the cortical breakthrough.

**Materials and methods:**

Experiments were conducted on ex vivo lamb shank by mimicking osteolytic bone tumours. The cortical breakthrough was exploited to induce hyperthermia inside the medullar cavity by delivering acoustic energy from a phased array HIFU transducer. MR thermometry data was acquired intra-operatory using the proton resonance frequency shift (PRFS) method. Active temperature control was achieved via a closed-loop predictive controller set at 6 °C above the baseline. Several beam geometries with respect to the cortical breakthrough were investigated. Numerical simulations were used to further explain the observed phenomena. Thermal safety of bone heating was assessed by cross-correlating MR thermometry data with the measurements from a fluoroptic temperature sensor inserted in the cortical bone.

**Results:**

Numerical simulations and MR thermometry confirmed the feasibility of spatio-temporal uniform hyperthermia (± 0.5 °C) inside the medullar cavity using a fixed focal point sonication. This result was obtained by the combination of several factors: an optimal positioning of the focal spot in the plane of the cortical breakthrough, the direct absorption of the HIFU beam at the focal spot, the “acoustic oven effect” yielded by the beam interaction with the bone, and a predictive temperature controller. The fluoroptical sensor data revealed no heating risks for the bone and adjacent tissues and were in good agreement with the PRFS thermometry from measurable voxels adjacent to the periosteum.

**Conclusion:**

To our knowledge, this is the first study demonstrating the feasibility of MR-guided focused ultrasound hyperthermia inside the medullar cavity of bones affected by osteolytic tumours. Our results are considered a promising step for combining adjuvant mild hyperthermia to external beam radiation therapy for sustained pain relief in patients with symptomatic bone metastases.

## Background

Bone is a common site for metastases in advanced cancers, the third most frequent after the lung and liver, occurring mainly in breast and prostate cancers [1–3]. Pain from bone metastases is frequent, severe in advanced cases and limits quality of life [4–6]. Since the underlying mechanism is not completely understood, pain management remains a challenge, and treatment is often palliative.

Radiotherapy (RT) is one of the standard palliative treatment modalities effective for painful bone metastases and acts by controlling the progression of the metastatic disease [7, 8]. However, pain recurrence after RT treatments was observed in 23-25% of the cases [9].

After treatment, 50% of the patients reported pain relief after 4 weeks [10], and 24% of the patients suffered a pain relapse at 3 months [11]. As recent developments in systemic treatments have allowed significant improvements in survival outcomes of patients with metastatic disease, and as re-irradiation may be challenging [12], the development of alternative and more effective treatment modalities [9–13] seems crucial to improve treatment response and outcomes, even in a metastatic setting.

In addition to traditional approaches (surgery, radiotherapy and chemotherapy), thermal therapies are nowadays being increasingly recognized as treatment options for primary cancers, as well as for metastases [14]. In thermal ablation, heating of the tumour tissue to temperatures of about 55–60 °C for short time periods (typically less than 2 min) causes thermal denaturation of proteins and cell membranes, and results in cell death within minutes (via coagulative necrosis) or hours (via apoptosis). In mild hyperthermia, target tissue is heated to around 41–43 °C for longer time periods (typically between 30 and 60 min) with the purpose of thermal sensitization, i.e., to enhance the effects of radio- and chemotherapy [15, 16] or for local drug delivery [17].

To increase tissue temperatures, usually electromagnetic or ultrasound energy, such as radiofrequency waves (RF), microwaves (MW), laser, or high intensity focused ultrasound (HIFU), is directed at the target tissue. An interstitial or intracavitary antenna can be used for RF/MW, a fiber-optic probe is inserted into the tumour for laser therapy, while in cryoablation tissue freezing is performed via cryoprobes inserted into the tissue [14]. Though minimally invasive, these techniques are difficult to apply to bone tumours, since they require the insertion of a heat (or cold) source directly into bone tissue. In contrast, HIFU is a non-invasive technology allowing the delivery of acoustic energy with millimetre accuracy, deep inside the body [18, 19]. For some specific applications, endocavitary applicators of therapeutic ultrasound have also been developed [20].

Since the 1990s, HIFU therapy has been coupled to magnetic resonance imaging (MRgHIFU) for targeting, intra-operatory control of sonication [21], and early assessment of radiological changes in tissue [22]. Near real-time MR temperature monitoring based on the proton frequency resonance shift (PRFS) enables feedback for the automated control of the ultrasound beam to achieve precise spatially-uniform heating [23, 24]. Clinical application of MRgHIFU has been demonstrated for liver, breast, prostate and brain tumours [25–28].

Clinical application of MRgHIFU in the field of bone pathology is challenging, as the ultrasound absorption rate of cortical bone is high and only a small amount of the energy passes through the cortex, thus preventing the ablation of tumours within the intramedullary space of intact cortical bone [29]. Therefore, HIFU application had been limited to the direct ablation aiming palliation of pain caused by superficial lesions, as the energy at the bone surface increases rapidly and damages the highly innervated periosteum [9, 30]. However, recently it has been shown that modulation of treatment parameters (low frequencies, increased acoustic energy levels and number of sonications) permits heating beyond the cortex [31–33].

HIFU ablation on bone produced coagulative necrosis of bone marrow, cortex and surrounding tissues in the short term but did not induce fractures or affect elastic stiffness Bone healing and intramembranous bone regeneration was reported at one month after treatment [32, 34–36]. Pain relief is also achieved by the control of the metastatic microenvironment. In particular, the ablation of osteoclasts, major contributors to local acidosis, results in pain relief by reducing local acidosis—a factor acting in afferent nociceptors [37–42]. However, the ablative treatment must be carefully monitored, since the temperature inside the bone is much higher than the one measured in the periosteal region. A study of HIFU interaction with ribs noted a large difference between the PRFS temperature estimation in soft tissue adjacent to the rib and the readings of a gold standard fluoro-optic thermometer inserted in the medullar cavity [41].

Presently, HIFU ablation is recognized as an efficient approach to relieve pain in patients refractory to RT and has been used for the treatment of osteoid osteomas and primary bone malignancies [30, 33, 43–48].

Besides tissue ablation, HIFU can induce mild (non-ablative) hyperthermia by delivering low sonication intensity over a long duration [23]. Hyperthermia in the range of 41 to 43 °C is well established as a radio and chemo-sensitizer for a wide range of malignant tumours [49–55]. It has been proven that hyperthermia inhibits the repair of DNA damage of malignant cells caused by RT [56]. Moreover, hyperthermia has been shown to decrease tumour hypoxia and increase tumour perfusion [57], induce tumour apoptosis and enhance immune effector cell proliferation [58, 59].

Clinically, hyperthermia of superficial tissue is passively achieved using a warm fluid flow (Alba ON 4000, Alba hyperthermia System, Via Adriano Olivetti, 24, 00131 Rome Italy), while deep regional hyperthermia is achieved using a localized heat source generated by either an interstitial or intracavitary antenna of RF/MW [53, 60]. Since for bone tumours the insertion of a heat source directly into bone tissue is technically difficult, extracorporeal sources of electromagnetic field have been investigated. The BSD-2000 system (Pyrexar Medical, Salt Lake City, UT, US), containing an external array of RF antennae, has been used to treat a variety of cancers [26, 61], but to our knowledge has not yet been reported for the treatment of bone tumours. A recent randomized phase III clinical trial was the first one to study adjuvant hyperthermia combined with RT for the treatment of painful bone metastases [62]. In this study, hyperthermia was delivered using a pair of parallel circular electrodes situated on opposite sides of the body (Thermotron RF-8 system). A phantom study using the same RF system [63] reported that bone absorbs the electromagnetic energy more intensively than surrounding tissues, complicating the model-based planning of hyperthermia. However, the implementation in the clinical routine of the combined hyperthermia-RT treatments using commercially available RF devices still raises significant technical challenges, mainly due to the creation of hot spots in normal tissues and to the lack of accuracy in the spatial control of the hyperthermia. Thus, the development of novel approaches based on MRgHIFU, such as the present one, is anticipated to overcome these issues for hyperthermia delivery and thus to improve the overall performance of combined RT-hyperthermia treatments.

In a preclinical study evaluating drug delivery enhancement using rabbit thighs, MRgHIFU with a closed-loop temperature controller was used to generate mild hyperthermia in healthy intact tissue at a bone and muscle interface [24].

**Fig. 1:**
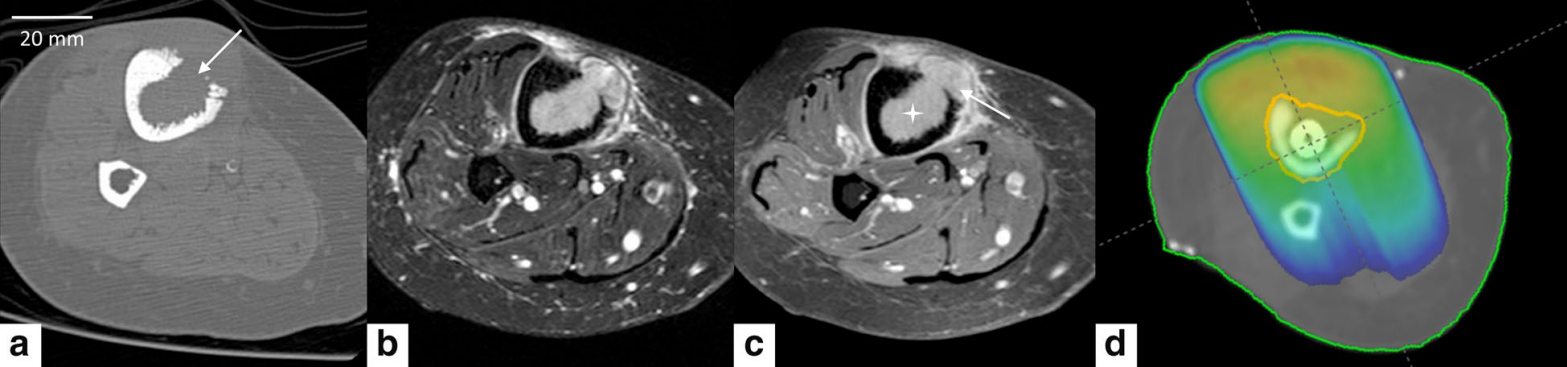
Pictorial illustration of bone metastasis in the right tibia of endometrial adenocarcinoma in 66 years old woman and the pain palliation RT dosimetry. CT in axial bone kernel (**a**) showed cortical interruption (see arrow). Axial MRI in Short-TI Inversion Recovery (STIR) T2w (**b**) and T1 fat saturation after gadolinium injection (**c**) showed, in addition to the cortical interruption, the medullar filling with tissue mass (see star) and a periosteal reaction (see arrow). Antero-posterior 6-MV photon fields dose distribution (**d**) in color-wash showing the 95% isodose line of the same patient (prescription dose 30 Gy in 10 fractions)

In this study, we investigate HIFU-induced hyperthermia inside the medullar cavity of an ex vivo bone model mimicking osteolytic lesions. A typical clinical situation, serving as ground truth for our model, is shown in Fig. 1, corresponding to a bone metastasis in the right tibia treated with palliative RT. In order to enable the delivery of the HIFU beam inside the tumour, we propose to exploit the natural acoustic window provided by the cortical breakthrough, that is, a sector of cortical bone destroyed by the tumour and replaced with a soft tissue mass, as produced by some osteolytic bone lesions. The sonication is further coupled with automatic stabilization of temperature at a prescribed level. To our knowledge, this is the first study using MRgHIFU for mild, non-ablative hyperthermia to demonstrate of MRgHIFU-based mild (non-ablative) hyperthermia demonstrating the possibility of inducing uniform temperature elevation inside the osteal medulla for several tens of minutes, without thermal risk for the adjacent cortical bone and the surrounding tissue.

## Materials and methods

### Instrumentation

The procedure was performed using an MR-compatible phased array HIFU transducer (Imasonic, Besançon, France). The transducer was a spherical cap of radius 130 mm working at a frequency of 1.031 MHz and powered by a 256-channel beam-former (Image Guided Therapy, Pessac, France). The most central 172 elements of the transducer were active yielding an aperture of 120 mm (f-number = 1.08). The applied acoustic power was 60 W, which corresponds to 432 W/cm^2^ at the focal point according to a study by Saletes and al. [64]. The duty cycle was adapted on-the-fly to the measured tissue temperature, as detailed below. In these conditions, the wave propagated essentially in linear regime with a focal negative peak pressure of -3.6 MPa and a focal positive peak pressure of 4 MPa. Hynynen K. [65] demonstrated this pressure range does not produce inertial cavitation.

Experiments were carried out on a 3T MRI scanner (Prisma Fit, Siemens) with an 11 cm diameter receive loop coil. Geometry of the tissue sample and HIFU transducer were verified on a high resolution T1-weighted 3D MR sequence with spatial resolution and a T1 contrast optimized for visualization of cortical bone, bone marrow, muscle and tissue mimicking gel (gradient echo, TE = 2.46 ms, TR = 5.36 ms, flip angle = 10°, bandwidth [BW] = 390 Hz/pixel, slice thickness = 0.8 mm, isotropic voxel).

Temperature elevation in tissue mimicking gel and muscles was measured using the PRFS method. As already reported, PRFS method does not apply to the cortical bone nor the bone marrow [66, 67]. Here a single slice, segmented gradient echo EPI GRE-EPI sequence was acquired, either perpendicular or parallel to the bone axis, with a spatial resolution of 1 mm × 1 mm × 4 mm and a temporal resolution of 1.6 s. The imaging parameters were: a 1-2-1 binomial spectrally selective pulse train to exclude the fat signal, TE = 8.46 ms, TR = 70 ms, EPI factor = 7, flip angle = 15°, BW = 698 Hz/pixel, acquisition matrix 128 × 128, FOV = 128 × 128 mm, number of averages = 1 and phase encoding direction parallel to the B_0_ field. The current TE value appeared to be the best compromise between magnitude SNR, phase CNR and partial volume mitigation at bone interface.

B_0_ drift correction is mandatory over a long period of MR acquisition. Therefore, PRFS thermometry data was corrected for background phase drift using a small unheated ROI positioned in the muscular tissue unheated by the HIFU beam.

### Experimental model

An anatomic model mimicking osteolytic bone tumors was used, consisting of drilled and mechanically excavated lamb tibia in situ. The procedures were performed in a bath of 0.9% NaCl physiologic serum, to avoid the penetration of air.

**Fig. 2:**
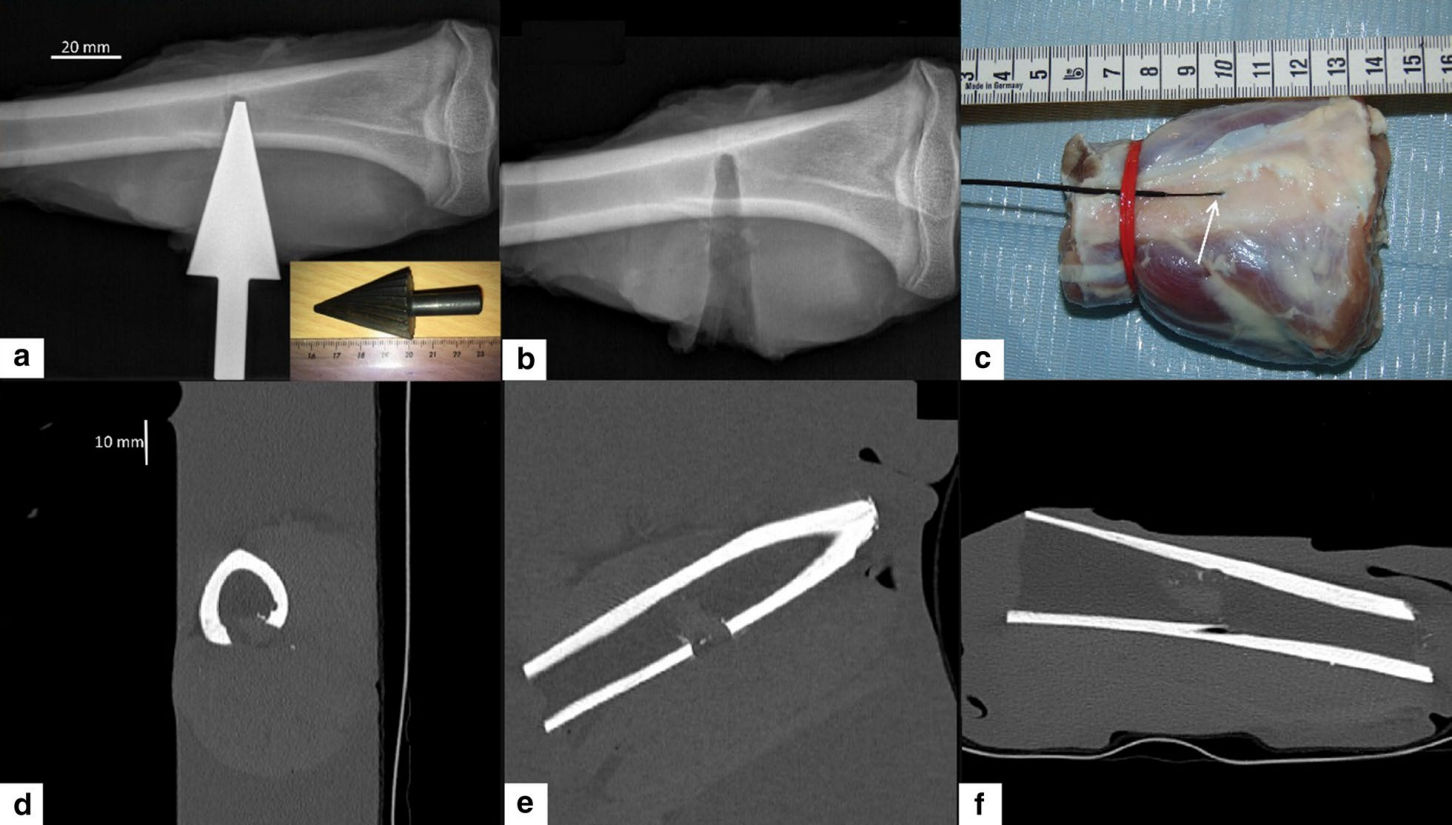
Geometry of the ex vivo samples. **a** Conventional radiograph of a lamb bone with in situ conical drill, also shown in a photograph; **b** Conventional radiograph after removal of the drill; **c** Photograph of the anterior side of the sample illustrating the insertion; **d**–**f** Illustrative 3D CT MPR images of a similar sample. Graphical distance scales are provided

**Fig. 3:**
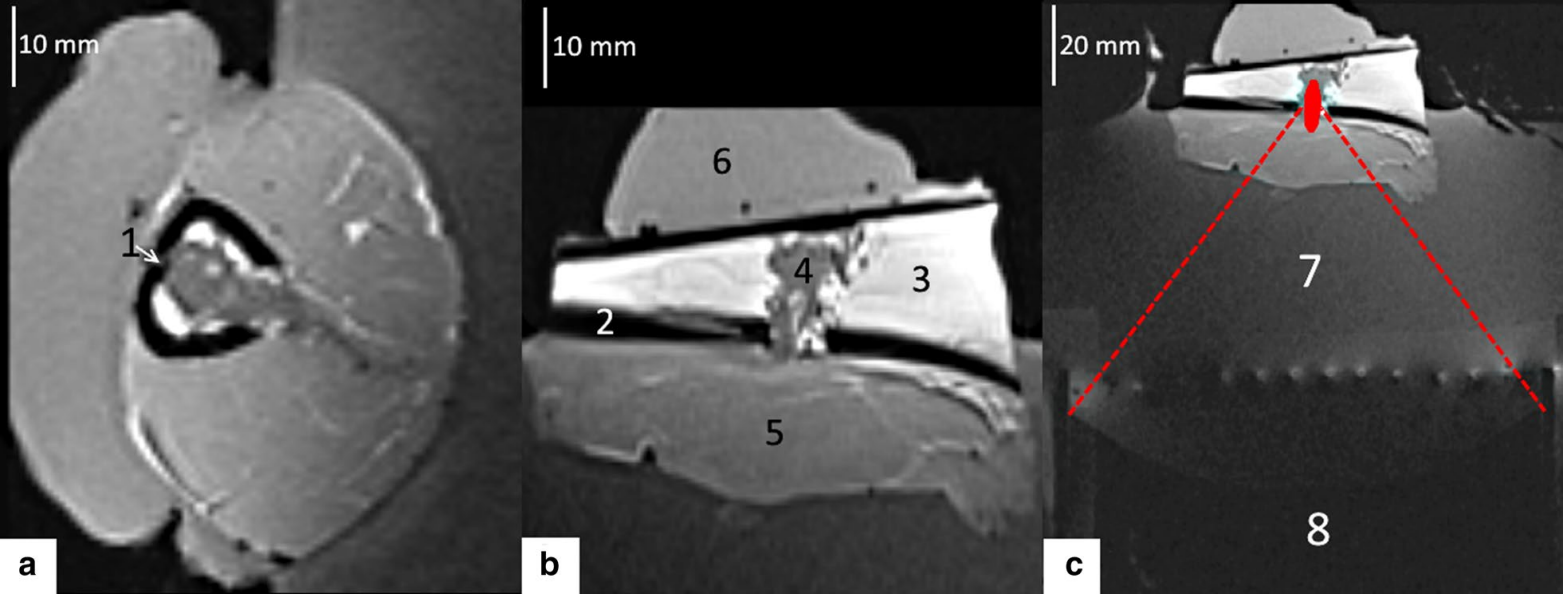
T1w 3D MPR MR images of the experimental setup, with a sample positioned for MR-guided HIFU hyperthermia. **a**, **b** Magnified images perpendicular and parallel to the tibia, respectively, square FOV 128 mm. **c** Axial image illustrating the HIFU transducer and the targeting of the lesion. Embedded legend: 1: intracortical tip of optical fiber, 2: cortical bone, 3: medulla, 4: tissue mimicking gel, 5: muscle, 6: ultrasonic gel, 7: degassed water, 8: spherical transducer

We started with a thin incision on the superficial aponeurosis and muscles in front of the desired target. A deeper incision was made over the intermuscular septus to expose the tibial shaft. A marking point on the surface of the bone was made with a scalpel. This marking point was further used as the starting point for bone drilling with an initial cylindrical bit of 6 mm diameter. In a second step, a larger cylindrical bit (10 mm diameter) or a conical bit (Fig. 2a) were used to reproduce an osteolytic lesion. The effect of the drilling was controlled interleaved with conventional radiography (Fig. 2b). Curettage was performed in order to create a bone medullar cavity by removing the fat. After drilling the cortical bone, an orthopedic surgical 10 mm curette was used to excavate the medullary fat, to remove it and thereby to produce lacuna mimicking a lytic bone tumor. Cavity size was dependent on the bone diameter, the latter was measured retrospectively to be between 7.6 and 15.5 mm (Fig. 3a, b). Medullar cavities were filled with tissue-mimicking gel [68, 69], exhibiting thermo-acoustic properties similar to soft tissue. The gel was composed of water (80.1%), glycerol (11.2%) added to adjust the acoustic velocity, agar (3%) added to adjust the stiffness and SiO_2_ (5.59%) added to adjust the acoustic attenuation [68]. Ramnarine et al. [69], reported the gel properties to be very similar to human tissue, namely: speed of sound = 1541 ± 3 cm s^−1^, attenuation = 0.5 ± 0.03 dB cm^−1^ MHz^−1^ over a frequency range of approximately 3–10 MHz, and density = 1054 ± 1 kg m^−1^. The mixture was liquid above 50 °C and set as a gel in a less than one minute after intra-cavitary injection via a 16G needle. Standard ultrasonic gel was applied at the interface between the cavity and muscles. The procedure was finished by sewing the different teguments with a surgical knot using non-absorbable 2–0 suture (PolysorbTM, Covidien, Dublin, Ireland).

The prepared lamb shank was degassed for 30 min under vacuum to prevent exogenous cavitation nuclei and near field scattering. Then, the shank was placed in the aperture of the MR loop coil and acoustically coupled with the HIFU transducer, see Fig. 3c. The tibia was lying approximately horizontal and the sample was rotated to approximately align the cortical breakthrough with the main direction of the HIFU beam (vertical). Fourteen experiments were conducted on six bone specimens (Table 1). A single focal point was positioned (1) in front of the medullar cavity, (2) in the plane of the cortical breakthrough or (3) inside the medullar cavity. Each scenario was tested with preset power sonication and controlled hyperthermia at + 6 °C above baseline, maintained within the tissue-mimicking gel for 12 min.

**Table 1:** Overview of the full series of experiments

Sample number	Hyperthermia experiment	Medullar cavity long axis [78]	Mean temperature elevation (°C)	Controller precision (°C)	Delivered energy (kJ)
1	#1	10.0	5.82	0.26	7.87
1	#2	10.0	6.22	0.20	7.69
2	#3	16.1	6.71	0.41	8.92
2	#4	16.1	6.92	0.31	8.59
2	#5	16.1	5.91	0.41	10.0
3	#6	12.9	6.42	0.23	10.07
4	#7	14.1	6.20	0.11	12.17
4	#8	14.1	5.69	0.29	10.85
5	#9	14.9	6.18	0.18	6.93
5	#10	14.9	5.72	0.18	5.74
6	#11	10.9	6.30	0.15	10.50
6	#12	10.9	5.87	0.20	7.47
6	#13	10.9	6.05	0.12	7.79
6	#14	10.9	6.32	0.61	8.07

The mean temperature elevation corresponds to the centre of medullar cavity during the steady state regime, to be compared with the prescribed elevation of + 6 °C

### Gold standard thermometry

MR thermometry in the cortical bone is limited, mainly due to the lack of signal as the T2* is very short. Thus, in order to assess the method’s safety with respect to bone heating, the temperature in the cortical bone was monitored using a gold standard method, namely a fluoroptic temperature sensor (0.9 mm diameter, STF-5, Luxtron, Santa Clara, CA, USA) ensuring a precision of 0.1 °C for relative temperature measurement. To this purpose, the cortical bone was drilled nearly parallel to its long axis with a thin bit (1 mm diameter), at a 10° angle relative to the tangential direction, until approximately 2/3 of the cortical thickness. Standard ultrasonic gel was injected in the resulting “tunnel” for optimal thermal coupling and finally the sensor was inserted into (Figs. 2c, 3a). PRFS thermometry measurements in soft tissue adjacent to the periosteum were cross-correlated with the fluoroptic measurements inside the cortical bone. Since fluoroptic sensors will not be available during clinical application, these correlations will allow the extrapolation of PRFS measurements to the cortical bone region for safety assessment.

### Predictive temperature controller

Extensive work has been reported for temperature control of ultrasound based thermal therapy [23, 24, 70, 71]. Some commercial products including Sonalleve^®^ and TUSLA^®^ devices from Profound Medical (2400 Skymark Avenue, Unit, Mississauga, ON L4 W 5K5, Canada), have standard capabilities for automatic feedback. These systems are insufficient for the current application due to the prolonged temperature elevation delay inside the cavity after HIFU sonication. The feedback control should predict temperature elevation for several minutes in advance.

A predictive temperature controller was designed, which automatically adjusted the acoustic energy deposition. A temperature elevation inside the medullar cavity is obtained via two mechanisms. The first mechanism is the direct absorption of focused ultrasound beam around the focal point, located in the tissue-mimicking gel or in the skeletal muscle in front of the cortical breakthrough. The second mechanism consists of the so-called “acoustic oven effect”, namely a high absorption of the post-focal acoustic energy on the internal facet of the cortical wall, followed by passive heat flow centripetally inside the cavity. The second mechanism, simulated quantitatively in the next section, yields a temporal lag between the acoustic power command and the temperature response at the location of the focal point, on the order of 1 min and increasing with the size of the cavity.

Practically, heat conduction requires a time interval to propagate the temperature elevation from the internal cortical facet to the actual location of the focal point. The designed temperature controller aims to stabilize the temperature elevation at the location of the focal point according to a prescribed level. The propagation delay between the input signal and the output function of a regulated system is a well-known problem impacting the process stability. This problem can be alleviated either using a long dwell time of the regulation loop (defined as the time interval after which the controlled parameter is modified), or a predictive physical model to anticipate the system response. Since increasing the dwell time would impact the precision, which is incompatible with the rather narrow range of acceptable temperature elevation for mild hyperthermia, we opted for a predictive approach.

In order to keep the acoustic intensity invariant during active periods of HIFU energy delivery, the controlled parameter was chosen to be the sonication duty cycle δ, varying in the interval [0, 1]. The amount of energy, *E*, delivered to the tissue during the acquisition of one MR thermometry map with temporal resolution $\Delta t$ is expressed as:

1 $$E = \delta \cdot P \cdot \Delta t,$$

where *P* is the measured acoustic power, calibrated in the free field using the radiation force balance. The hyperthermia treatment starts with the initial condition $\delta = \delta_{0}$. The temperature controller is activated when the temperature elevation in a small ROI around the focus reaches an out-of-noise threshold defined here at 1.5 °C, which was 5 to 10 times the noise standard deviation of the input function.

**Fig. 4:**
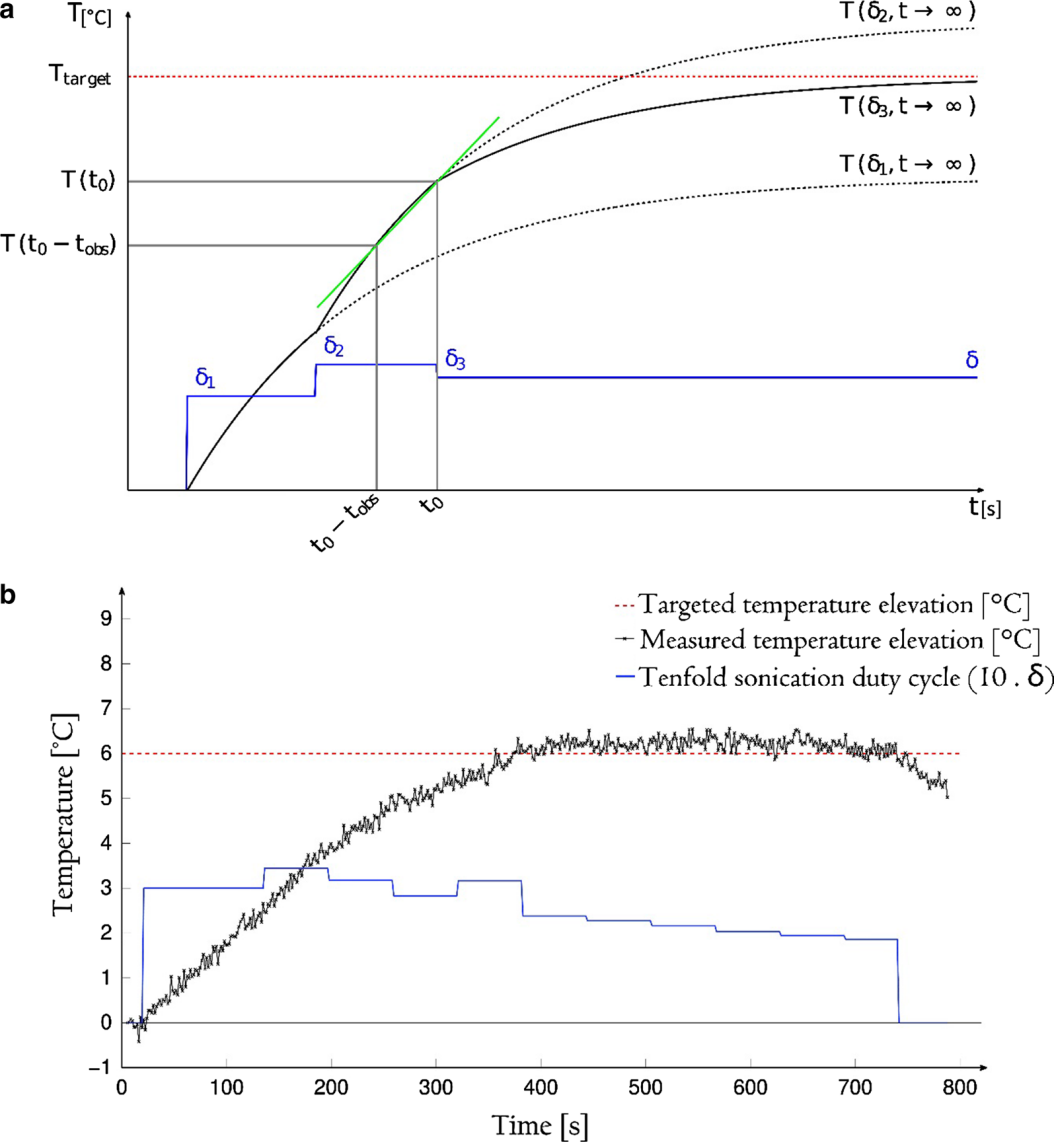
Theoretical and experimental performance of the predictive temperature controller. **a** Exponential projections calculated at each time point of observation. The average slope of the curve is used to estimate the asymptotic values $T\left( {\delta, t \to \infty } \right)$, which are further used to adjust the new duty cycle of sonication. **b** Measured average temperature in a 13-pixel ROI inside the medullar cavity (axial PRFS thermometry) and the applied duty cycle versus time. Same experiment as shown in Figs. 2 and 6

The temperature elevation above the physiological baseline at the focal point location, using a sonication duty cycle $\delta$, is denoted as $T\left( {\delta , t} \right).$ The physical principle is to estimate the asymptotic level of temperature elevation that would be reached if the parameter $\delta$ was kept constant, $T\left( {\delta , t \to \infty } \right)$, see Fig. 4a. Ideally, this estimation should match the prescribed level of temperature elevation, denoted as $T_{target}$. Otherwise, the parameter $\delta$ should be increased or decreased, depending on the position of the asymptote below or above the prescribed level. Thus, the update of the parameter $\delta$ after $n$ intervals of dwell time is calculated using the following master equation:

2 $$\delta_{n + 1} = min\left\{ {\delta_{n} \cdot \frac{{T_{target} }}{{T\left( {\delta_{n} , t \to \infty } \right)}},\;1} \right\}.$$

The asymptotic level of temperature elevation $T\left( {\delta , t \to \infty } \right)$ is estimated from the measured temperature values within a temporal sliding window of observation. The length of the observation window $t_{obs}$ was empirically chosen (here, 32 s) to be half of the dwell time of controller (here, 64 s), in order to achieve a compromise between robustness against measurement noise when computing the numerical derivative, and reaction speed of the temperature controller. The estimation of $T\left( {\delta , t \to \infty } \right)$ at any time point $t_{0}$ during hyperthermia is based on approximating the further evolution of the temperature elevation at the focal point as a mono-exponential function (Fig. 4a). The details of the estimation are presented in Appendix 1.

Theoretically, the sonication duty cycle can vary between 0 and 1. However, the effective range of 0.1 to 0.9 to accommodate the time request for hardware communication, switching delays, and feedback power meters. Since the master equation of the temperature controller [2] is multiplicative, we have chosen as starting value for this parameter the geometric mean between the two extremes, namely $\delta_{0} = 0.3$. Practically, every half second the beam former emits a pulse length of $0.5 \cdot \delta$ (s), followed by an off period of $0.5 \cdot \left( {1 - \delta } \right)$ (s).

### Numerical simulation

A Matlab^®^ (MATLAB 9.2, MathWorks, Inc., Natick, Massachusetts, USA) numerical simulation estimated the time lag between the application of sonication and focal point peak temperature elevation. The simulation also suggested the optimal focal point position relative to the bone cavity. The bone was modelled as a cylinder of radius Rc. The radius of the cortical bone (Rc) in the axial plane was varied in the range of 6 to 10 mm, and the focal point position was prescribed at various loci relative to the center of the breakthrough in the range (− R_c_, R_c_). To mimic an osteolytic tumour, the cavity in the bone diaphysis was simulated as the intersection between the bone cylinder and a cone of aperture 72°, whose axis was normal to the cylinder axis, and whose apex was on the cylinder axis. A single element transducer of focused ultrasound was considered, with diameter 120 mm, radius 130 mm, and operating frequency 1 MHz.

The physical interaction HIFU beam/bone is a complex phenomenon [72, 73]. The analytical description of the underlying physics is considered beyond the scope of this report. Instead, we shall further employ a semi-empirical model that is consistent with the observed thermal effects. The following aspects were considered: (1) the acoustic properties are significantly different in cortical bone as compared to the adjacent soft tissue, by a factor of 2 in term of celerity and a factor of 3.6 in term of mechanical impedance; these differences are generating strong boundary conditions (reflection, refraction and, above the critical incidence angle, evanescent waves); (2) due to its solid structure, the bone supports the generation and propagation of shear waves in addition to the longitudinal compressional wave; (3) the thickness of the studied cortical bone (i.e. a few millimeters) is comparable with the wavelength of compressional and shear waves, and also comparable to the penetration depth of evanescent waves.

An evanescent wave can be described as a near-field wave, which is traveling along a boundary, so that the pressure and particle motion amplitudes decay exponentially as a function of the actual depth into the surrounding media. Multiple boundary reflections can occur as a wave travels forth and back inside the cortical bone, e.g. “wave guide” effect. Petrusca et al. [41] described near isotropic isotherms around near field sonicated ribs, that is, thermal patterns rotationally invariant with respect to the direction of the incident HIFU beam. In our numerical computation, we modelled these phenomena as a global averaging operator applied to the acoustic energy distribution within the segment of the cortical bone crossing the conical HIFU beam.

The thermal effect of the absorbed acoustic waves was computed as a three-step process. The technique is similar to the approach described by Salomir [71]. First the complex pressure field and the acoustic intensity generated by the transducer was calculated. Then the effect of the cortical bone was taken into account by extracting the acoustic intensity along the 3D cortical bone internal facet exposed to the HIFU beam, and applying an averaging operation on the respective surface (mean intensity). This step models the mechanical energy redistribution mentioned above and observed by [41], also called herein “acoustic oven effect”. Finally, heat diffusion during HIFU sonication was simulated by iterative convolution with a Gaussian kernel [71], considering both the cortical and tumoral absorption. The details of the computation are provided in Appendix 2.

## Results

### Numerical simulations

**Fig. 5:**
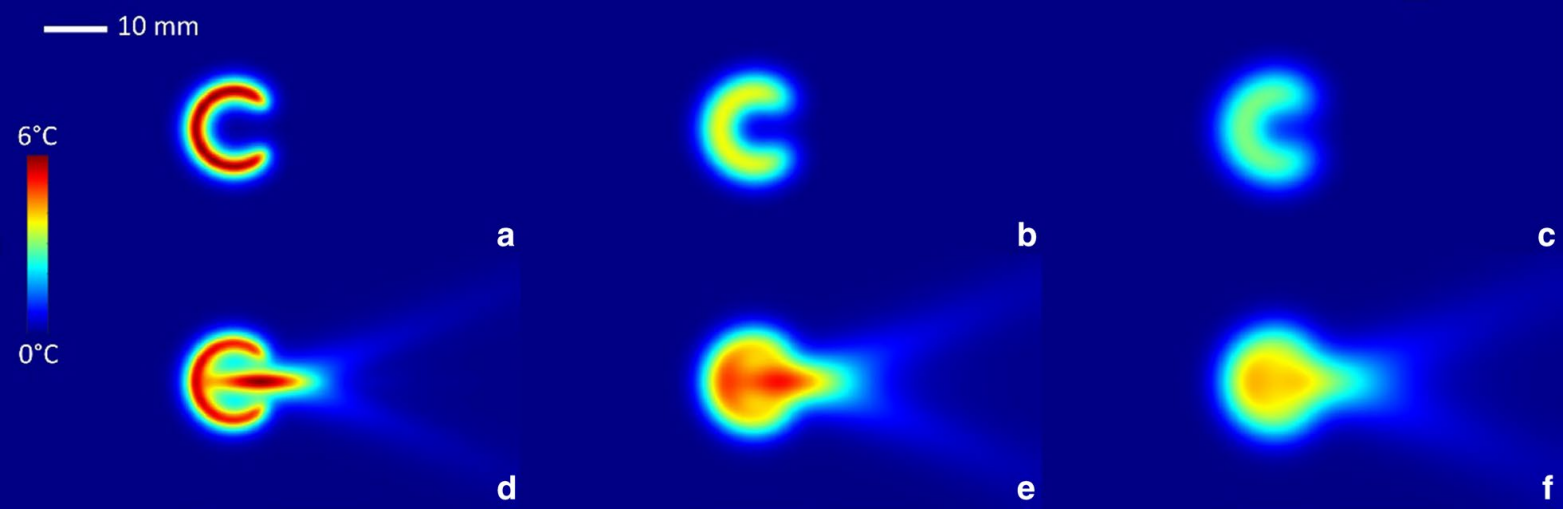
Numerical simulation of the impulse response function of one osteolytic lesion, sonicated with the focal point symmetrically positioned in the center of the cortical breakthrough, axial bone diameter 12 mm. **a** Temperature elevation at the end of a short HIFU sonication isolating only the cortical source, followed by a free evolution of **b** 40 s and **c** 60 s. **d** Temperature elevation at the end of a short HIFU sonication considering the cumulated source of heating followed by a free evolution of **e** 40 s and **f** 60 s. Temperature color bar and graphical distance scale are provided

**Fig. 6:**
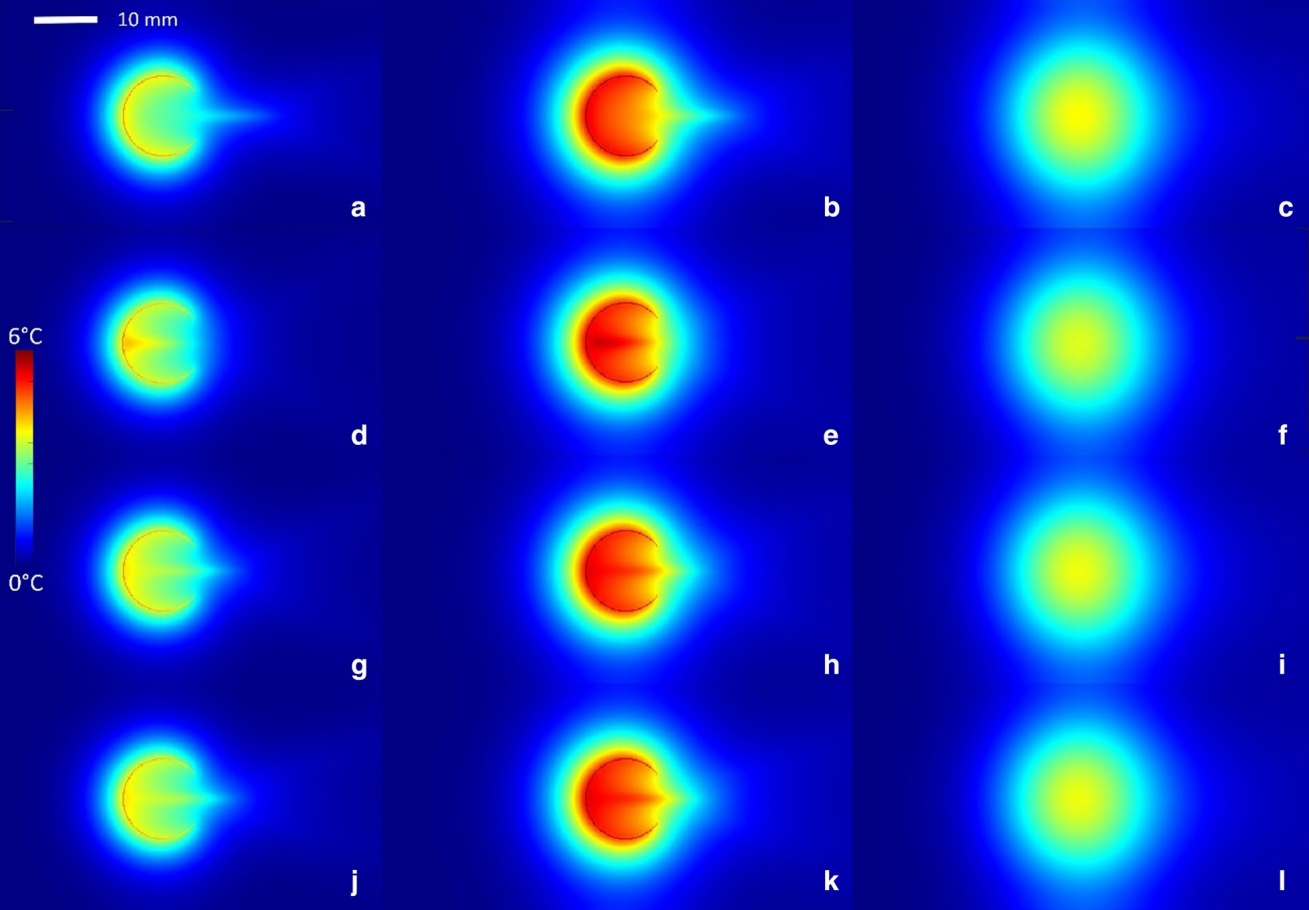
Numerical simulations of long sonication (480 s), for a bone of 14 mm internal diameter. The time points of the first, second and third column are 240 s, 480 s and 540 s, respectively. The focal point is shifted along the acoustic axis with respect to the centre of the breakthrough by − 7 mm, 7 mm, 0 mm and 0 mm top down respectively. The three first rows illustrate the computational results for a single element transducer (f = 130 mm, d = 120 mm) while the last row correspond to the computational results considering the natural focusing of the phased array (f = 130 mm, d = 120 mm) employed in the experimental study

The results of the simulations are presented in Figs. 5 and 6. The cortical source of thermal energy is illustrated in Fig. 5a, and the cumulated source of cortical and tumoral thermal energy is illustrated in Fig. 5d. Post-sonication tissue cooling was simulated similarly to Eq. (A9) by nulling the $\alpha$ and $\beta$ coefficient. The relaxation process following a short sonication (i.e. impulse response function) is illustrated in Fig. 5b, c for the cortical source, and in Fig. 5e, f for the cumulated source.

The analysis of the impulse response, shown in Fig. 5, indicated that the main contribution to the intra-cavitary heating is the “acoustic oven effect” on the cortical border. However, because of the cortical breakthrough, the isotherms would remain asymmetric. The other source of energy, namely the direct heat deposition around the focal point, positioned at the site of the breakthrough, had the role to bridge the circumferential isotherms. The symmetry of the resulting isotherms was influenced by several factors, namely the size of the breakthrough, the actual position of the focus and the ratio of ultrasound absorption between the cortical bone and tumoral tissue. In general, the balance of these factors under the typical conditions of an osteolytic tumour yielded approximately circumferential isotherms. The heat diffusion tended to attenuate the residual inhomogeneities, if any, as seen in Fig. 5f.

The analysis of long duration sonication confirmed the generation of circumferential isotherms under the effect of the double source of heating and the heat diffusion (Fig. 6). When the focal point was shifted inside the cavity, a “hot spot” was detected at the proximal internal facet of the cortex, as the two heating sources were locally cumulated (Fig. 6g). This configuration may yield a thermal risk. Reciprocally, when the focal point was set at the center of the breakthrough, the temperature map evolved more rapidly towards a uniform spatial profile (Fig. 6e). Finally, shifting the focal point opposite to the cavity (Fig. 6b) had less influence of the local cortical heating. However, when the size of the breakthrough was small, this condition yielded hot spots at the edges of the breakthrough. Overall, we confirmed hereby the optimal positioning of the focal point at the center of the breakthrough, using an ideal single element applicator or a phased array with equivalent F-number (frames g–l).

The described numerical model permitted the estimation of the time lag ($\varepsilon$, expressed in s) between the end point of HIFU sonication and the time point where the temperature elevation reaches a maximum at the focal point location as a function of two parameters: the bone section radius $R$ (expressed in mm) and the focal point offset with respect to the breakthrough plane, denoted as $H$ (expressed in mm). Because the time lag is due to the “acoustic oven effect”, only the heat generation on the internal surface of the cortical bone was considered in the calculations. The time lag was described with very good accuracy by a quadratic function (average error 10 s):

3 $$\varepsilon = \mathop \sum \limits_{m,n = 0}^{2} c_{mn} H^{m} R^{n} ,$$

where $c_{00} = 236\;{\text{s}},\;c_{10} = 7.83 \;{\text{s/m}}, c_{01} = 65\;{\text{s/m}},\;c_{20} \; = \;1.54 \;{\text{s/m}}^{2} ,\;c_{11} \; = \; - \;4\;{\text{s/m}}^{2} ,$ and $c_{02} = 7.94 \;{\text{s/m}}^{2} .$ A graphical illustration is provided in Additional file 1. The time lag rapidly increased with the radius of the bone section. Therefore, the dwell time of the automatic temperature controller needs to be increased for larger lesions.

When the focal point offset $H$ was set to zero, which is the optimal condition according to the previous observations, Eq. [3] simplifies to a second order polynomial of *R.* Given that the average radius of our samples was 6 mm, the time lag was calculated to be 126 s. Further details on the numerical results are provided in Additional file 1.

### Procedural findings

Geometrically, the ex vivo model was very similar to the clinical conformation, see for comparison Figs. 1a and 2d. The described experimental model was systematically free of air bubble contamination at tissue interfaces, as demonstrated by high resolution 3D MR imaging (Fig. 3). The most challenging step appeared to be the uniform curettage of the medullar cavity, since this step was lacking imaging guidance. Subsequently, the medullar cavity might contain residual fatty tissue, further impacting the PRFS MR thermometry near the internal facet of the cortical bone.

HIFU targeting of bone pseudo-tumor was feasible in all specimens. Low energy pilot sonications permitted visualization of the focal spot on MR thermometry axial or parallel to the bone, and consequent application of electronic steering until the main beam axis was centered on the cortical breakthrough. No acoustic obstacle or beam distortion by tissue interfaces was noted to occur from the injection of tissue mimicking gel, the local dissection of the muscle, the mitigation of the tissue dissection using ultrasonic gel, or the sewing of the different teguments with surgical wire.

### MR thermometry and automatic temperature control

The pixel-wise temporal standard deviation of MR thermometry in the tissue mimicking gel was on average 0.2 °C. Figure 4b illustrates an example of the experimental temperature elevation at the focus versus time, using the automatic temperature control, together with the actual parameter $\delta$ applied per dwell time interval. The duty cycle gradually converged to a value of approximately 0.18 (60% of the initial one), corresponding to 11 W average acoustic power, which is 79 W/cm^2^ focal acoustic intensity, and corresponds to 1.5 MPa positive peak pressure and 1.3 MPa negative peak pressure. A steady-state regimen, defined by an absolute offset of less than 0.2 °C between the actual temperature elevation and the predefined target, was obtained in 260 s in average (min: 160 s, max: 370 s). The steady-state temperature elevation calculated as the spatio-temporal average inside a 13 pixel ROI during the steady-state was 6.16 ± 0.23 °C in 14 procedures, compared to the pre-defined temperature elevation of 6 °C. The average relative error was therefore inferior to 4%. Given the consensual hyperthermia range between 41 and 43 °C, corresponding to a 95% confidence interval of ± 1 °C, the average precision of temperature control should be at least as low as 0.5 °C.

**Fig. 7:**
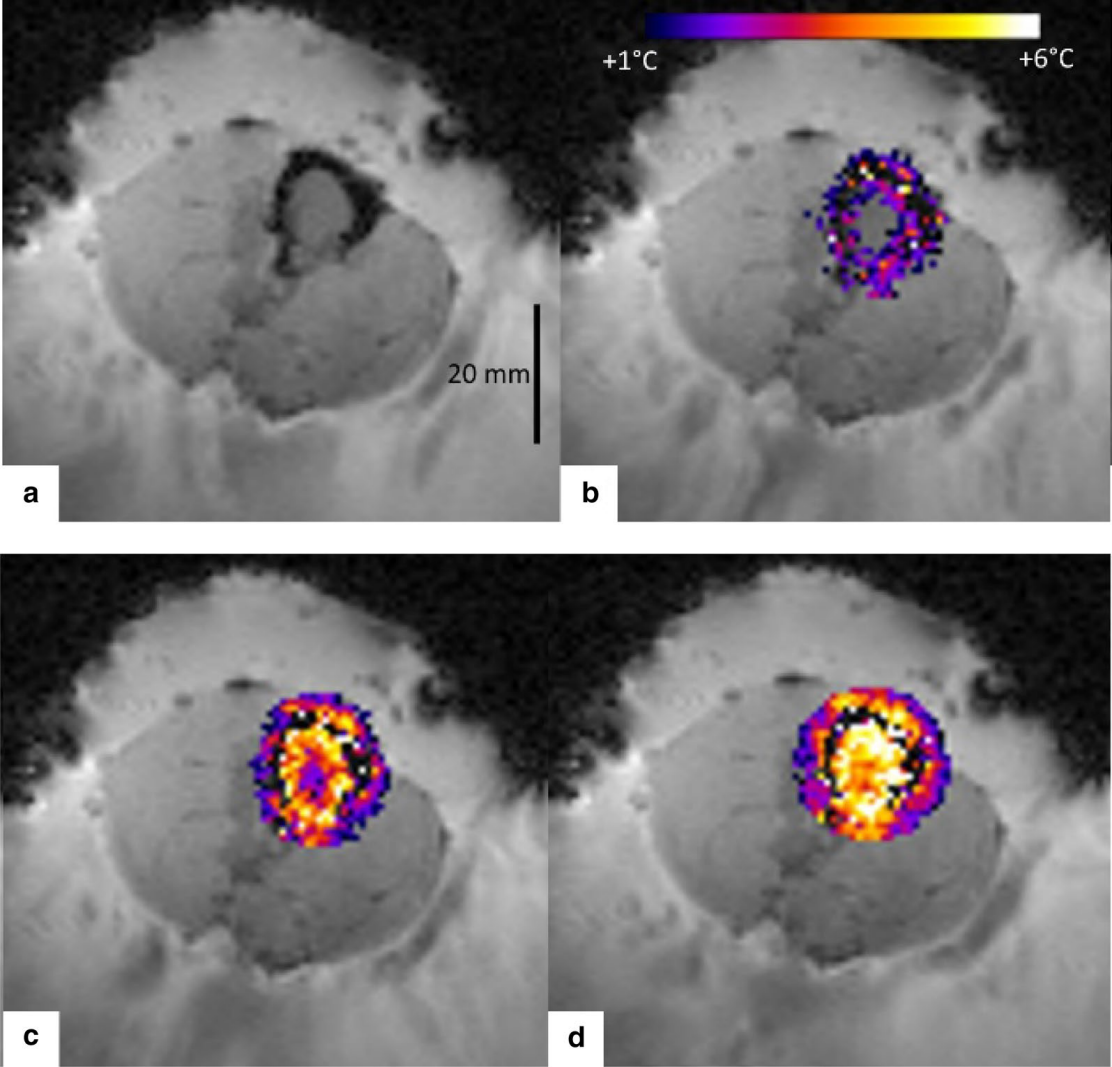
2D MR thermometry maps during MRgHIFU hyperthermia of a bone tumor mimicking model, in a plane perpendicular to the bone long axis and with the focal point positioned in the center of the cortical breakthrough. Data are shown: **a** Prior to HIFU sonication, **b**–**d** 80 s, 160 s, and 320 s after the onset of HIFU sonication, respectively. Note the circumferential temperature elevation of the internal facet of the cortical bone (“acoustic oven effect”) and the evolution of the thermal build-up inside the medullar cavity towards a uniform distribution

**Fig. 8:**
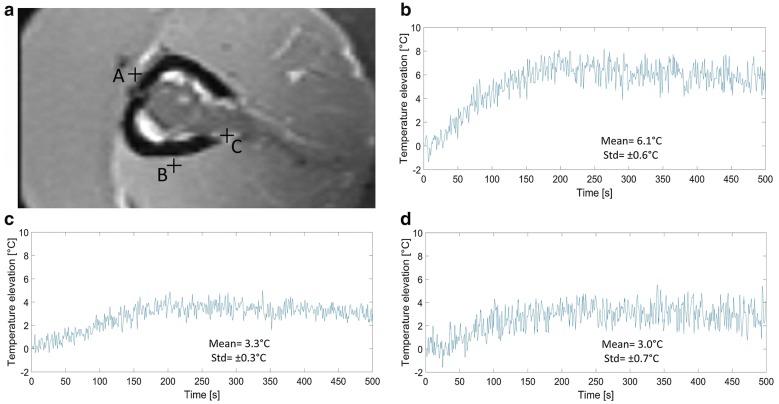
PRFS thermometry data obtained in three specific voxels adjacent to the periosteum. **a** High resolution T1w image perpendicular the bone. Note the chosen locations A, B and C. **b**–**d** Temperature elevation versus time for locus C, A and B respectively

**Fig. 9:**
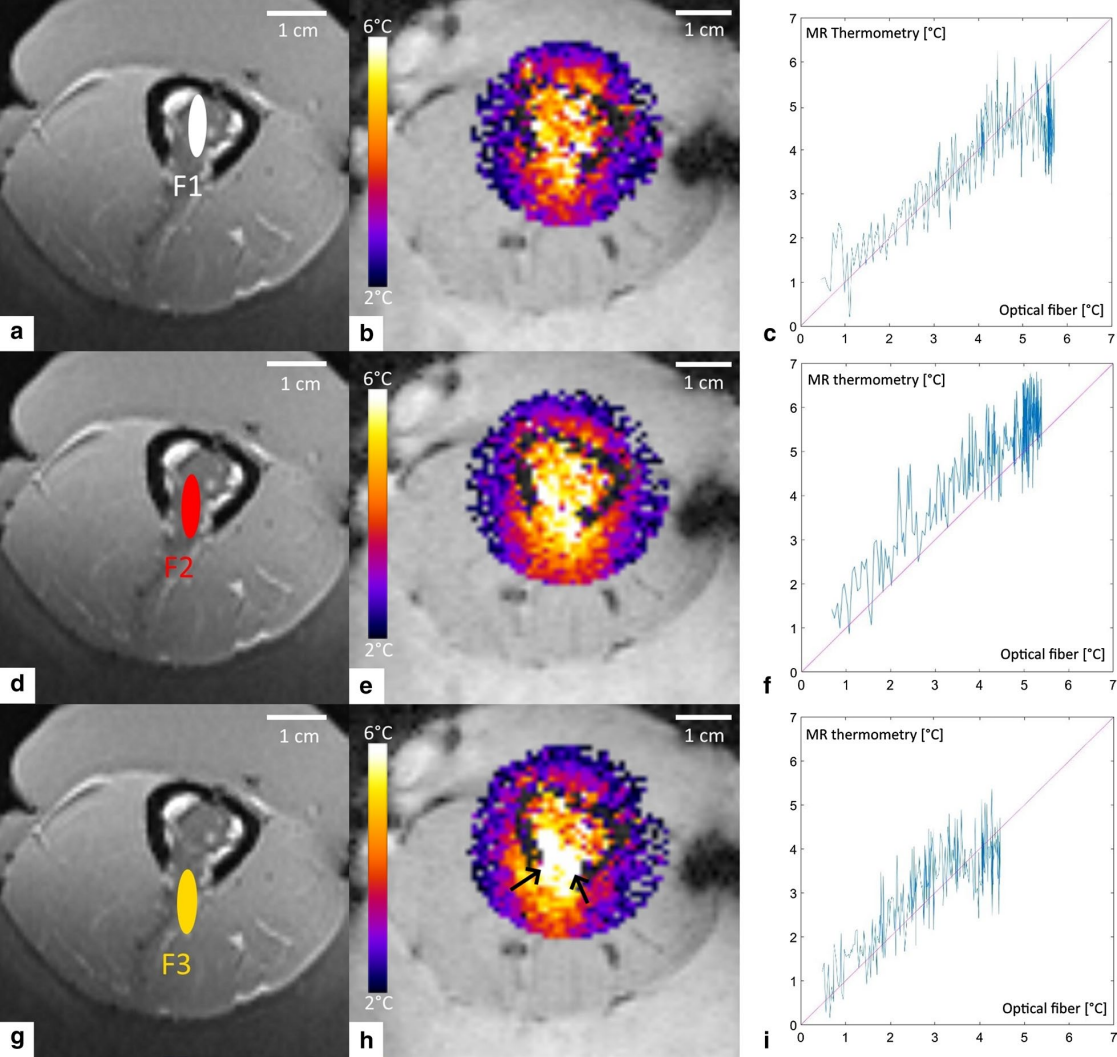
Experimental investigation of focal point positioning: **a**–**c** 5 mm inside the medullar cavity; **d**–**f** in the plane of the cortical breakthrough; **g**–**i** 5 mm in front of the medullar cavity for a given sample. The columns show (left to right, respectively): the position of the focus, the axial temperature elevation map after 400 s of sonication, the cross correlation between the PRFS thermometry in soft tissue adjacent to the bone and the fluoroptical measurements inside the cortical bone

The measured temperature maps demonstrated that the thermal build-up inside the medullar cavity can be approximated by concentric isotherms originating on the cortical bone (Fig. 7b–d), as a joint effect of superficial averaging of acoustic energy on the internal facet of the cortical bone and of heat diffusion, the so-called “acoustic oven effect”. In frame (d) it is clearly visible that the local energy deposition by the focal point at the breakthrough site is bridging the circumferential isotherm. Figure 8 provides further insights into the spatio-temporal distribution of the temperature. The plots indicate that the temperature elevation at representative locations in soft tissue (adjacent to the periosteum lateral and posterior to the acoustic axis, adjacent to the breakthrough edge) was confined in the range 50% to 100% of the + 6 °C target. Figure 9 illustrates the comparative results for a variable depth (− 5, 0 and 5 mm) of the focus with respect to the plane of cortical breakthrough, on the same sample, all other parameters remaining unchanged. Positioning the focus in the plane of the cortical breakthrough (Fig. 9e) yields nearly perfect thermal patterns. Deeper positioning (inside the medullar cavity) yielded preferential heating of the opposite cortex wall where was inserted the fluoroptical sensor. For each focal depth (− 5, 0 and 5 mm), the sensor read the following highest values of temperature elevation 4.5 °C, 5.4 °C and 5.7 °C. More proximal positioning (in front of the breakthrough) yielded enhanced heating at the lateral edges (see black arrows in frame 9h). Plots of the cross correlation between PRFS thermometry data and sensor data are provided in Fig. 9c, f, g. PRFS thermometry data obtained in a voxel adjacent to the periosteum immediately behind the focal point and preserving sufficient MR signal, was found to be in very good agreement with the sensor data (mean offset 0.75 °C, min − 0.5 °C, max 1.7 °C), for each geometry of the HIFU beam.

In terms of temperature controllability and absence of thermal risk for the exposed cortical bone, the optimal positioning of the focal point was therefore found to be the geometrical center of the cortical breakthrough. These findings are consistent with numerical simulations (section C). The temperature controller performance was stable for each focal depth. Only one global maximum was observed on the time temperature curves and no oscillations were depicted. When the focal point was in the optimum position, the observed overshoot was minor, if any, and lower than 1 °C.

## Discussion

To our knowledge, this is the first study of hyperthermia inside the bone medullar cavity directly sonicated with HIFU. Until recently, most studies focused on the palliation of painful bone metastases via HIFU ablation of the periosteum, since heating of the medullar cavity is technologically challenging due to the high absorption rates of the acoustic energy by the cortical bone [44–46, 74]. However, more recent studies demonstrated that the medullar cavity could be heated indirectly by varying the HIFU acoustic parameters (frequency, number and intensity of sonications) [31–33]. In a recent report, Bucknor et al. [31] investigated the effect of different sonication parameters on the average depth of ablation following MRgHIFU of a swine femoral model in vivo. Unlike our model of osteolytic bone metastases, the sonicated bone was intact, that is, a cortical breakthrough was not performed, and the duration of sonication did not exceed 40 s. They reported that at equal energy, shorter higher-power sonications produced deeper ablation sites compared to long duration, lower-power sonications. These findings are most likely related to the blood perfusion and complex (non-linear) interactions between the focused ultrasound beam and the cortical bone. Considering these potential effects, we have chosen to adapt the duty cycle of the sonication, while keeping invariant the acoustic power for the entire hyperthermia treatment. The temporal lag between the acoustic source command and the intra-medullar temperature response may yield periodic oscillations of the controlled temperature when using proportional-integral-derivative controller (preliminary data not shown). Our predictive controller suppressed the fluctuations and demonstrated a range of accuracy clearly sufficient for clinical application.

In a pioneering work, Staruch et al. [24] studied MRgHIFU-based hyperthermia at the muscle-bone interface of in vivo rabbit thighs, based on the implementation of a proportional-integral (PI) temperature controller. They showed that mild hyperthermia enhanced drug delivery in heated versus unheated bone marrow. However, there are several differences between their study and our proposed approach. First of all, our method allows the temperature monitoring and control inside the medullar cavity, whereas in [24] temperature control was achieved only at the muscle-bone interface, while the temperature in the bone and medullar cavity was extrapolated through simulations. However, unlike our study, these simulations were not validated through gold-standard thermometry, thus the safety of the procedure needs further validation. Moreover, the temperature controller implemented in the present study is predictive, thus allowing anticipation and better control of delayed heating effects occurring due to the beam interaction with the bone internal facet. It allowed the attainment of a uniform temperature elevation inside the medullar cavity, closely matching the prescribed temperature. Further improvement of the controller’s performance may be obtained by analysing the experimental impulse response function in situ using MR thermometry, prior to the effective hyperthermia sonication, that is, by estimating the case-specific temporal lag between the acoustic source command and the intra-medullar temperature response. Our approach capitalizes on the “acoustic oven effect” to achieve uniform heating inside the medullar cavity, while keeping the focal spot fixed. Conversely, beam-steering was used in [24] to obtain a circular heated region. When targeting is achievable with mechanical positioning of the transducer, the current application simplifies the transducer design and avoids the secondary effects stemming from beam steering, such as the appearance of grating lobes.

The “oven effect” has been mentioned mostly in the context of RF ablation [75, 76], where, due to its low thermal conductivity, bone is seen as an insulator, trapping the heat and resulting in heating augmentation. Beside the similarity of thermal patterns, we are exploiting here a different phenomenon, renamed “acoustic oven effect”. Petrusca et al. [41] studied HIFU beam interaction with the ribs in the context of liver tumor ablation, revealing a nearly isotropic heat distribution around the external and internal facets of the ribs, when the focal spot was placed in the liver position (behind the ribs). We hypothesized here that the physical interactions between HIFU and the bone diaphysis can be modeled mathematically by applied a superficial averaging of the incident HIFU energy over the axial perimeter of the sonicated cortical bone. Unlike intact cortical bone, the existence of the cortical breakthrough yielded in our study HIFU interactions occurring on the inner facet of the cortical bone.

This effect provides the base for the most important achievement of this study, namely the delivery of uniform isotherms within the osteolytic lesion, with a size of the order of 1 cm, using only a fixed focal point position, in other words, without using electronic beam steering for volumetric heating. The optimal position of the focal spot was found to be in the plane of the cortical breakthrough, confirmed by both experimental and numerical simulation.

The mechanism of bone lytic metastases pain is heterogeneous, complex and mediated by neuro-immune factors. Moreover, this phenomenon is independent of the size of the metastasis [77]. However, a minimum breakthrough diameter is required to enable the HIFU beam penetration inside the cavity and this minimum diameter is several times the acoustic wavelength. Thus, the maximum size of the eligible tumor should be defined taking into account the potential risk for adverse effects.

Due to the “acoustic oven effect”, the average acoustic power required in our study was very low and the hardware requirements were therefore minimal for the HIFU transducer, of the order of 10 W. This value is to be compared with reported 120 W for MRgHIFU thermal ablation of bone metastases [3].

Cortical bone is not directly accessible to standard PRFS MR thermometry, and alternative techniques are currently under development [66]. When applicable, the PRFS method is advantageous for fast acquisition, linearity and tissue independent calibration. In this study, PRFS temperature monitoring of adjacent soft tissue was demonstrated to be a very good substitute for cortical bone temperature monitoring, according to the clinical needs and criteria, under the present conditions of mild hyperthermia. Here, a slow heating rate was applied as compared to the intrinsic time of heat conduction. Moreover, our temperature measurements in the soft tissue adjacent to the bone suggest the absence of thermal risk for neighboring tissue if the hyperthermia is conducted according to the conditions of this study. Thus, the proposed treatment is expected to yield no adverse effects on the bone or the surrounding healthy tissue, neither intra- nor post-operatory. This finding may not be valid in the case of a rapid heating rate, as for instance in thermal ablation.

The main envisaged application of our proposed method is bone hyperthermia as adjuvant therapy combined with RT, for the palliative treatment of painful osteolytic bone metastases. This would allow the reduction of the RT dosage, thus enabling the repetition of the treatment if necessary. Towards the end of clinical application, the safety of the procedure and the absence of irreversible damage to the bone or its adjacent tissues have been warranted by the proven stability of the temperature controller. Moreover, the uniformity of the temperature rise inside the medullar cavity ensures an optimal treatment efficacy for the whole lesion. A worthwhile future extension of our work would be the study of bone hyperthermia through an intact cortical bone, thus enlarging its application domain and potentially benefitting patients with intra-medullary disease.

However, it could be argued that the use of HIFU thermal ablation in the palliation of painful bone metastases has already been validated by several clinical studies, and it does not require the supplementary step of RT. Nevertheless, our technique provides two main advantages. First, we demonstrated controlled uniform heating inside the medullar cavity, thus allowing the treatment of tumours therein, whereas the clinical application of HIFU ablation has only been proven for the superficial layer of the bone. Secondly, the technological risks of mild hyperthermia are lower than those of ablation. Ablation is performed by short HIFU sonications of relatively high intensity, heating tumor tissue at temperatures between 55 and 80 °C in order to induce coagulation. In contrast, in mild hyperthermia, temperature elevations are only a few degrees, but need to be maintained for longer periods of time (30–60 min). The higher temperatures required by ablation imply higher risks concerning the undesirable treatment of nearby healthy tissues, thus requiring a precise control of the heated locations. Meanwhile, since mild hyperthermia is performed for longer time periods, it benefits from the effects of heat diffusion, which contribute to creating uniform temperature elevation profiles with minimal displacement of the focal spot. However, temperature control techniques for hyperthermia need to take into account the long-term effects of heat diffusion, with the main challenge being the maintenance of tissue temperature in a narrow range. A common risk of both ablative and hyperthermic sonication is near-field heating, stemming from long term heat diffusion to nearby tissues in the case of hyperthermia, and from thermal doses cumulated in nearby tissues from successive HIFU sonications of tumor locations in the case of ablation.

Several limitations of this study should be mentioned. First, the ex vivo model did not include tissue perfusion phenomena. Perfusion rate is also dependent on tissue temperature and therefore subject to dynamic changes during the hyperthermia procedure. However, the closed-loop control of temperature is expected to manage this additional variability, given the slow temperature evolution over minutes. Second, tissue motion may occur during long intervals of sonication, potentially impacting the accuracy of PRFS thermometry and the spatial precision of HIFU targeting. Device ergonomics and patient compliance with the proposed hyperthermia approach remain to be assessed with clinical trials. Alignment of the main axis of the HIFU beam with the cortical breakthrough may not be feasible for every target lesion, depending on patient anatomy and the specific region. We expect that a versatile robotized arm will be required to handle the transducer for optimal positioning, as suggested by Krafft et al. [78].

## Conclusion

This paper is the first to propose an approach for HIFU-based hyperthermia inside the medullar cavity of bones affected by osteolytic lesions. Stable uniform heating inside the medullar cavity was achieved via a predictive closed-loop temperature controller and validated through ex vivo experiments and numerical simulations. Our results capitalized on the dual heating mechanism inside the medullar cavity, through direct absorption around the focal spot and via the “acoustic oven effect” of the HIFU beam interaction with the bone internal facet. The method was proven to be safe with respect to heating of bone and adjacent healthy tissues. The current results are supporting the design of a prospective, interventional, single-center phase II study investigating the association of palliative single dose RT with a MRgHIFU-based HT for patients with painful bone metastases.

### Supplementary information

**Additional file 1: Figure S1.** Contour of the temporal lag (units: s) between sonication command and local extrema of temperature at the location of the focal point, as a function of cortical bone radius and focal point offset with respect to the center of breakthrough. Dotted lines are numerically calculated values and continuous lines are the fitted contours using the Eq. (12).

## Data Availability

All data generated or analyzed during this study are included in this published article and its additional file.

## Appendix

### Appendix 1: Predictive temperature controller

The purpose of this section is to explain the estimation of $T\left( {\delta , t \to \infty } \right)$ from equation [2]. The estimation is based on the measured temperature values within an given a temporal sliding window of observation, whose length $t_{obs}$ is half the dwell time of the controller. Given a time point $t_{0} = 2 \cdot t_{obs} \cdot n$ along the thermal history of the focal point during hyperthermia, the further evolution of the temperature elevation at the focal point, using a constant $\delta_{n}$ parameter, is approximated as a mono-exponential function:

A1 $$T\left( {\delta_{n} ,t} \right) = T\left( {\delta_{n} ,t_{0} } \right) + A\left( {\delta_{n} } \right) \cdot \left[ {1 - e^{{ - \left( {t - t_{0} } \right)/\tau }} } \right] .$$

Here $\tau$ stands for the physical response time of the heated tissue, which depends on the cavity size and the focal spot position, as shown by our simulations. However, to enable through-sample comparison of experimental results, we used a fixed value $\tau = 150 s$ in this study, that is comparable but slightly higher than the average value derived from simulation (126 s, see “Results”). Meanwhile, the bone section is not perfectly circular, therefore the model might underestimate the real condition.

The asymptotic value of the temperature elevation is therefore:

A2 $$\mathop {\lim }\limits_{t \to \infty } T\left( {\delta_{n} ,t} \right) = T\left( {\delta_{n} ,t_{0} } \right) + A\left( {\delta_{n} } \right).$$

In order to compute $A\left( {\delta_{n} } \right)$ based on the temperature values measured during the observation interval $\left( {t_{0} - t_{obs} , t_{0} } \right)$, the derivative of Eq. (A1) at time $t_{0}$ was approximated as:

A3 $$\left. {\frac{dT}{dt}} \right|_{{t = t_{0} }} = \frac{{A\left( {\delta_{n} } \right)}}{\tau } \cong \frac{{T\left( {t_{0} } \right) - T\left( {t_{0} - t_{obs} } \right)}}{{t_{obs} }} ,$$

yielding

A4 $$A\left( {\delta_{n} } \right) \cong \frac{{T\left( {t_{0} } \right) - T\left( {t_{0} - t_{obs} } \right)}}{{t_{obs} }} \cdot \tau .$$

Thus, the asymptotic value of the temperature elevation at time $t_{0} = 2 \cdot t_{obs} \cdot n$ is obtained by combining Eqs. (A2) and (A4):

A5 $$T\left( {\delta_{n} , t \to \infty } \right) = T\left( {\delta_{n} ,t_{0} } \right) + \frac{{T\left( {t_{0} } \right) - T\left( {t_{0} - t_{obs} } \right)}}{{t_{obs} }} \cdot \tau .$$

Experimentally, the observed values $T\left( {t_{0} } \right)$ and $T\left( {t_{0} - t_{obs} } \right)$ were denoised by averaging 5 sampling points symmetrically around the actual measurement. If the measured temperature change during the observation time is lower than a predefined threshold, the experimental calculation of the time derivative from Eq. (A3) is considered too noise-dependent and the exponential model is not applied. Instead, the system is considered to be at steady state. That threshold was set here to 0.2 °C which represents 1.6 fold the standard deviation of the compound noise of the measured incremental temperature $T\left( {t_{0} } \right) - T\left( {t_{0} - t_{obs} } \right)$. Under the hypothesis of steady state, Eq. (A5) simplifies to:

A6 $$T\left( {\delta_{n} , t \to \infty } \right) = T\left( {\delta_{n} , t_{o} } \right).$$

### Appendix 2

The thermal effect of the absorbed acoustic waves was calculated as a three-step process:

1. The complex pressure field $p^{ *}$ was calculated using the Rayleigh integral on the active surface of the transducer $S$ sampled at 1:20 of the wavelength, taking into account the cylindrical symmetry in the plane parallel to the HIFU beam:
  A7 $$p^{ *} \left( {\rho ,z} \right) \propto {\iint }_{S} \frac{{e^{{ - ik\mid\overrightarrow {{r_{1} }} - \overrightarrow {{r_{2} }}\mid }} }}{{\mid\overrightarrow {{r_{1} }} - \overrightarrow {{r_{2} }}\mid }}dS_{n} ,$$
  where $\overrightarrow {{r_{1} }} \left( {\rho ,\theta ,z} \right)$ is the point of observation in the vertical plane, $\overrightarrow {{r_{2} }}$’ is a vector pointing to the surface of the transducer, and $k = 2\pi /\lambda$ is the modulus of the wave vector. The complex pressure field was further interpolated to a Cartesian 3D matrix with isotropic spatial resolution ${{\lambda }}/8$ and the acoustic intensity ($I$) was computed as its squared modulus: $I \propto \left| {p^{ *} } \right|^{2}$. The comparison between the normalized acoustic intensity along the axis of symmetry of the transducer using this code versus the O’Neil’s analytical approximation provided by K-Wave (1.2.1, University College London, Department of Medical Physics and Bioengineering, Gower Street London, WC1E 6BT, United Kingdom) showed negligible differences.
2. The effect of the cortical bone was taken into account by sampling the acoustic intensity along the 3D cortical bone internal facet exposed to the HIFU beam, modelled as an ideal surface denoted as ${{\varPsi }}$, and applying an averaging operation on the respective surface (mean intensity). This step models the mechanical energy redistribution mentioned above and observed by [41], also called herein “acoustic oven effect”. We defined therefore a shell source of thermal energy expressed as ${{\varOmega }} \cdot \delta_{D} \left( {d\left( { \vec{r},{{\varPsi }}} \right)} \right)$ where:
  - $d\left( { \vec{r},{{\varPsi }}} \right)$ is the 3d Euclidian distance between the point $\vec{r}$ and the ideal surface ${{\varPsi }}$ modelling the internal facet of the bone,
  - $\delta_{D}$ is the delta Dirac function
  - ${{\varOmega }}$ is the power of the shell source, calculated as ${{\varOmega }} = {{\alpha }} \cdot mean\left( {I\left( {\vec{r} \in {{\varPsi }}} \right)} \right)$ where ${{\alpha }}$ is the ultrasound absorption of the cortical bone [72].
  Because the delta Dirac function cannot be represented numerically, we discretized the shell source term as a one-pixel wide shell using the Heaviside function ${\mathcal{H}}$:
  A8 $${{\varOmega }} \cdot \delta_{D} \left( {d\left( { \vec{r},{{\varPsi }}} \right) } \right) \approx a \cdot {{\varOmega }} \cdot {\mathcal{H}}\left( {1 - d\left( { \vec{r},{{\varPsi }}} \right)} \right).$$
  The physical significance of parameter $a$ is the ratio between the average depth of penetration of the waves into the cortical bone and the size of the pixel used in the simulation.
3. Heat diffusion during HIFU sonication was simulated by iterative convolution with a Gaussian kernel $G$ of temporal resolution $\tau ,$ considering the cortical and tumoral absorption [71]:
  A9 $$\begin{aligned} T\left( {x,y,z, t + \tau } \right) & = T\left( {x, y, z, t} \right) \otimes G\left( {x,y,z,\tau } \right) \\ & \quad + a \cdot \alpha \cdot {{\varOmega }} \cdot {\mathcal{H}}\left( {1 - d\left( { \vec{r},{{\varPsi }}} \right)} \right) + \beta \cdot I\left( {x,y,z} \right) \\ \end{aligned}$$
  The β stands for the ultrasound absorption in tumoral tissue.
  According to literature, ${{\alpha }}$ = 6.9 dB/cm/MHz [79], and absorption of soft tissue or marrow is approximately 0.54 dB/cm/MHz [80]. Absorption in bone metastases is not reported explicitly in literature, but cancerous cells exhibit approximately 10% lower ultrasound absorption than normal cells [81]. Finally, we estimated the value of tumoral absorption $\beta$ = 0.5 dB/cm/MHz and the discretization parameter for the current resolution of simulation is $a$ = 3.

## Abbreviations

MR: magnetic resonance
HIFU: high intensity focused ultrasound
PRFS: proton resonance frequency shift
RT: radiotherapy
RF: radiofrequency
SNR: signal to noise ratio
CNR: contrast to noise ratio
