# Supplementary figures and images for: Mild hyperthermia by MR-guided focused ultrasound in an ex vivo model of osteolytic bone tumour: optimization of the spatio-temporal control of the delivered temperature
Source: J Transl Med. 2019 Oct 24;17:350. doi: 10.1186/s12967-019-2094-x (PMC6814062; doi:10.1186/s12967-019-2094-x)

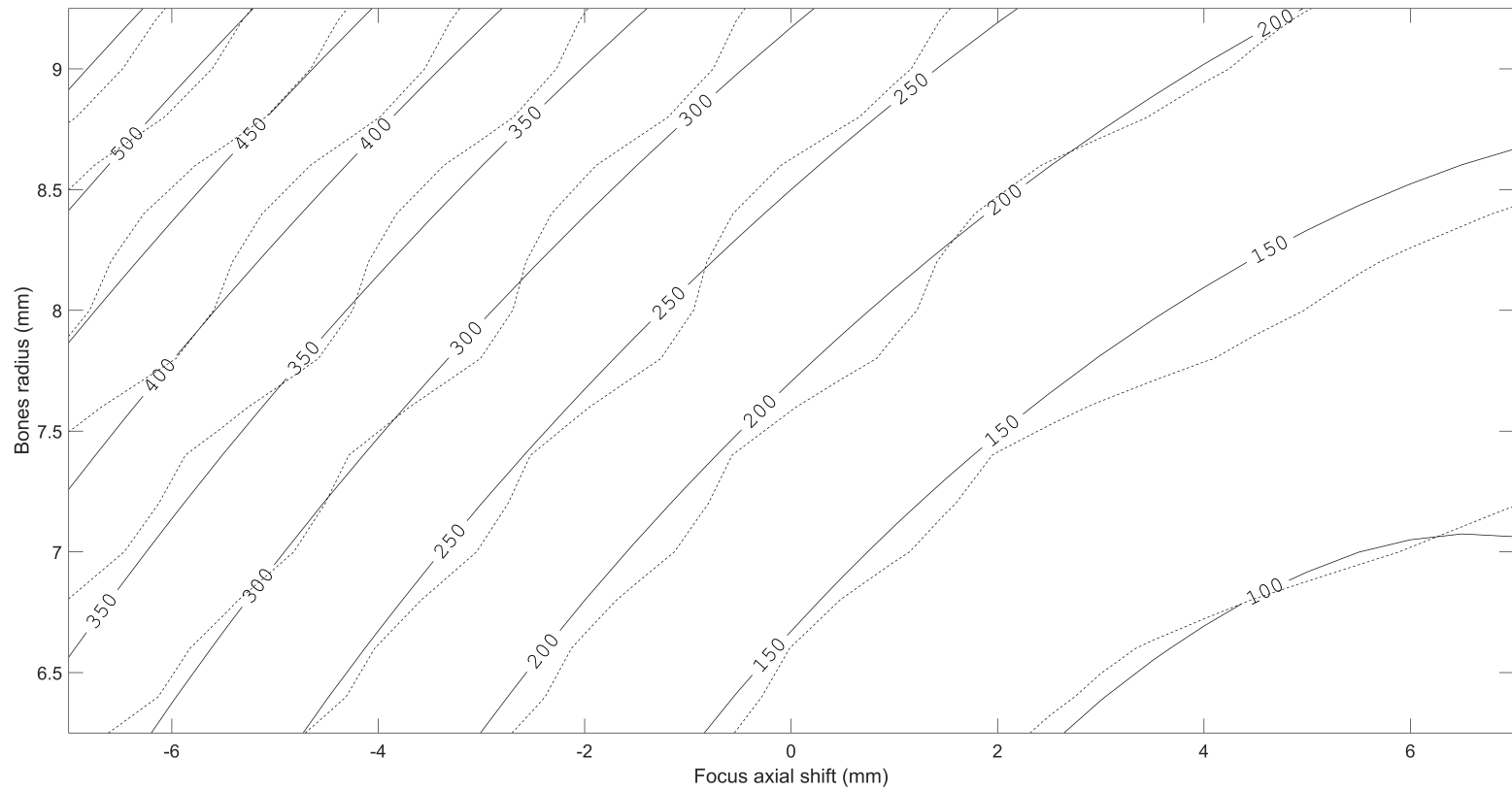

Supplement: Supplementary file 1 — Additional file 1: Figure S1. Contour of the temporal lag (units: s) between sonication command and local extrema of temperature at the location of the focal point, as a function of cortical bone radius and focal point offset with respect to the center of breakthrough. Dotted lines are numerically calculated values and continuous lines are the fitted contours using the Eq. (12). [file 12967_2019_2094_MOESM1_ESM.pdf]
